# Storm and tidal interactions control sediment exchange in mixed-energy coastal systems

**DOI:** 10.1093/pnasnexus/pgae042

**Published:** 2024-02-03

**Authors:** Ioannis Y Georgiou, Duncan M FitzGerald, Kevin C Hanegan

**Affiliations:** The Water Institute, New Orleans, LA 70122, USA; Department of Earth and Environment, Boston University, Boston, MA 02215, USA; Moffatt and Nichol, New Orleans, LA 70130, USA

**Keywords:** sediment transport, storms, sediment import, coastal inlets, backbarrier basins

## Abstract

Storms can have devasting effects on shorelines, causing flooding and the destruction of property and infrastructure. As global warming and the frequency and magnitude of tropical storms increase, barrier islands comprising 10% of the world's coast may undergo significant change caused by beach erosion, loss of dunes, and formation of washovers and tidal inlets. Understanding how storms affect sediment transport at tidal inlets is an understudied subject that directly influences barrier island erosional-depositional processes and long-term sediment budgets. This study models hydrodynamics and sediment transport at a conceptualized mixed-energy, mesotidal inlet system using 10 synthetic storm tracks. We investigate the provenance and the role of various storm characteristics and timing between the peak storm surge and high tide on sediment fluxes for different grain sizes. We find that most storms (38 of 40) cause a net import of sediment into the basin that is sourced primarily from the updrift and downdrift nearshore and secondly from the ebb-delta. Very little sediment comes from inlet channel scour. Cumulative (net) transport correlates well with peak significant wave height because wave height influences bottom shear stresses and sediment suspension on the ebb-tidal delta and in the nearshore. The duration of the storm surge also correlates with net transport because it controls the period of flood-directed currents. Our findings help explain the formation of flood deltas inside tidal inlets and the formation of sand shoals in backbarrier regions. Storm-induced enlargement of these deposits represents a permanent long-term loss of sand from barrier islands that will lead to erosion.

**Significance Statement**

Global warming and linked greater storminess are increasing sediment mobility and impacting barrier islands—tidal inlet sand budgets. Storms have a primary influence because they control net sediment transport at tidal inlets and erosional and depositional processes along adjacent barrier shorelines. We modeled 10 synthetic storm tracks at a mixed-energy, mesotidal tidal inlet with different timings of peak storm surge (40 modeled storm scenarios). Overwhelmingly, we find sediment is imported through tidal inlets during storms, which is sourced from areas immediately surrounding the inlet. Our results show tidal inlets are sediment sinks that remove sand from nearshore sand reservoirs, eventually leading to barrier island erosion.

## Introduction

While sea-level rise (SLR) changes coastal landscapes over centennial and millennial time frames, storms can significantly modify beaches and barriers over several hours, causing erosion and widespread destruction of property and infrastructure, extensive overwash, and the formation of new tidal inlets. Storms are also major drivers in redistributing sand reservoirs, a process that is particularly important at tidal inlets because these high-energy events can drastically increase the amount of sand entering tidal inlets (1). Barrier chains with their accompanying tidal inlets comprise ∼10% of the world's coastlines (2), and thus, storm processes at inlets are of interest to scientists and managers worldwide. Storms in conjunction with SLR are affecting both physical and ecological coastal environments by dramatically altering beaches, barrier islands, tidal inlets, and marshes through erosion and redistribution of sediment (3–8). For example, loss of salt marsh will change hydrodynamic characteristics at tidal inlets, such as tidal wave propagation in the backbarrier leading to flood or ebb-dominance (9–11), which in turn will control sediment fluxes and sediment budgets of these systems (12–15). Moreover, sedimentation in backbarriers can alter backbarrier hypsometry thereby changing tidal asymmetry and durations of ebb and flood-tidal currents (9). The nonlinear dependence of sediment transport on velocity dictates that slight asymmetries in velocities produce residual transport such that a basin will generally import sediment if tides are flood-dominant and export sediment if tides are ebb-dominant (9, 10).

Sediment fluxes through tidal inlets as well as in backbarrier tidal channels are integrative metrics from which net sediment budgets and transport mechanisms can be inferred. Unstable backbarrier systems will export sediment (due to marsh edge erosion and drowning), whereas stable systems will import sediment that preserves channel, flat, and marsh elevation during SLR (16). Subsequent work (17) highlighted additional factors that determine backbarrier stability including suspended sediment concentrations and external sediment sources. Modeling studies by Donatelli et al. (18, 19) have shown that marsh loss may decrease the capability of backbarrier basins to retain sediment, although this finding may be site specific. A later modeling study of Jamaica Bay, at the eastern end of Long Island, New York reported that the influx of marine sediment represents an important source of sediment to Jamaica Bay (20).

**Fig. 1.**
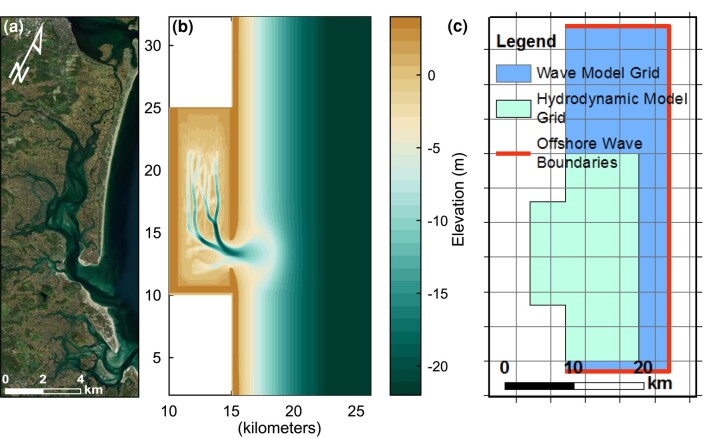
Conceptualized modeled basin. a) Aerial photograph mosaic of Plum Island Sound in northern Massachusetts, United States of America. b) Conceptual inlet-basin system Delft3D hydrodynamic, sediment transport, and morphologic model domain and initial bathymetry. c) High-resolution hydrodynamic and morphologic model grid, nested within coarse-resolution wave model grid with offshore wave boundaries. Initial bathymetry represents an approximately equilibrium condition reached after a 1.5-m amplitude semi-diurnal, sinusoidal tide and 0.25 m significant wave height, 6-s peak period wave imposed for 100 years of simulation time with a morphologic acceleration factor of 50 (model adopted from Hanegan et al. (22)).

**Fig. 2.**
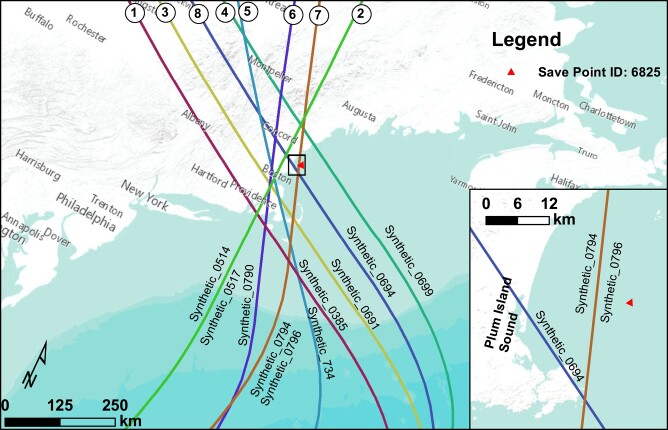
Tracks of synthetic storms used in this study that were compiled in the USACE NACCS (33). Simulated storm surge and wave parameters that were used to develop boundary conditions for the conceptual basin model were extracted at the red triangle from the USACE Coastal Hazards System (CHS) online interface (34) for *Base Conditions* storm simulations without tides.

Although tidal hydrodynamics and asymmetries are heavily influenced by the long-term evolution of tidal inlet morphology and basin hypsometry (9, 21, 22), in a regime of accelerating SLR, infrequent impacts from intense storms can contribute residual fluxes into or out of the basin, thereby influencing the morphologic trajectory of the basin (20, 21, 23–26). For example, sand import and deposition on flood deltas during large storms are important processes that help maintain tidal flats and build backbarrier marshes (27). Additionally, net sediment fluxes at marshes can be dominated by tidal processes or episodic storm events (17). Moreover, a modeling study of a barrier island chain along coastal Virginia reported that during intense storms, sediment is imported into basins, and the relative magnitude of sediment influx is a function of storm duration and surge magnitude as well as grain size (25). Their modeling results showed that mud and very fine sand were imported, whereas coarser sand was exported. Although this research suggests that intense storms may enhance the long-term sustainability of backbarrier bays (25), there is little understanding of the mechanisms during storms that cause the import of sediment. For instance, marsh platform inundation and delivery of sediment during storms relies on prolonged flooding, which can be influenced by the timing of high tides and storm peaks (28). Likewise, various storm characteristics (e.g. approach angle, track, intensity, speed, and size) can influence the magnitude and patterns of sedimentation (25, 28). Knowledge of the relative impact of these factors is important, as global warming is predicted to increase tropical cyclone activity in the North Atlantic (29). Despite the anticipated greater storm frequency and magnitude, as well as the ongoing accelerating SLR, both of which will modify tidal basin morphology and hydrodynamics (12, 21, 30), there has been little research on how future storm trends will impact basinal sediment fluxes. The influence of the relative phasing between tides and the storm surge, often with similar magnitudes along large portions of the east coast of the United States and elsewhere, has also received little attention. It is important to note that the import or export of sediment at tidal inlets can drastically affect the barrier island sand reservoirs leading to long-term erosion (e.g. Barataria Bay barriers, LA (26)) or deposition (e.g. East Frisian Islands (31); Plum Island, MA (32)). This study explores the impact of storms at a conceptual mixed-energy tidal inlet (Fig. 1) using a hydrodynamic and sediment transport model. We evaluate the role of various storm pathways (Fig. 2), characteristics, and timing between peak storm surge and high tide on inlet sediment fluxes (for different grain sizes), finding that most storms cause an import of sediment that will deplete nearshore and barrier sand reservoirs.

## Results

### Storm sediment fluxes

**Fig. 3.**
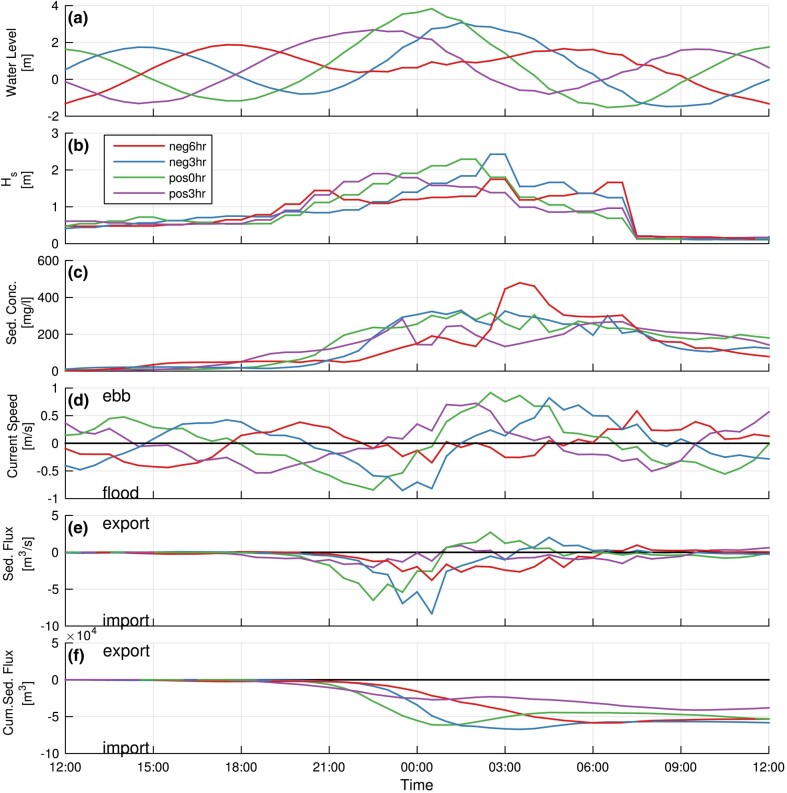
Model results for storm 0385 (see Fig. 2 for track) at the inlet for each of the four peak surge/tidal phasing scenarios (neg6hr, neg3hr, pos0hr, and pos3hr). a) Water level at the inlet superimposing the modeled storm surge from the NACCS study with a 1.5-m amplitude sinusoidal tide for various phases, b) significant wave height (Hs), c) total sediment concentration (sum of all grain size fractions), d) simulated water discharge through the inlet, e) total (sum of all fractions) sediment flux through the inlet, and f) cumulative sediment flux through the inlet during the storm period, indicating tidal basin import or export of sediment. Note that storm 0385 produced an import of sediment for all surge/tide phasing scenarios with the greatest import occurring when the peak surge preceded high tide by 3 h (neg3hr phasing).

Net sediment fluxes through the inlet for each storm and surge/tide phasing scenario were tracked by grain size fraction and provenance zone (e.g. Fig. 3). For all phasing scenarios, total sediment flux is first directed basinward (import) then seaward (export) with the storm impact (Fig. 3e), and the relative magnitude of the sediment import and export preceding and following the storm surge peak influences the net flux direction over the full simulation (Fig. 3f). Sediment transported through the inlet during tidal cycles before and after storm passage is orders of magnitude less than during the storm such that the influence on net transport is negligible (Fig. 3f).

**Fig. 4.**
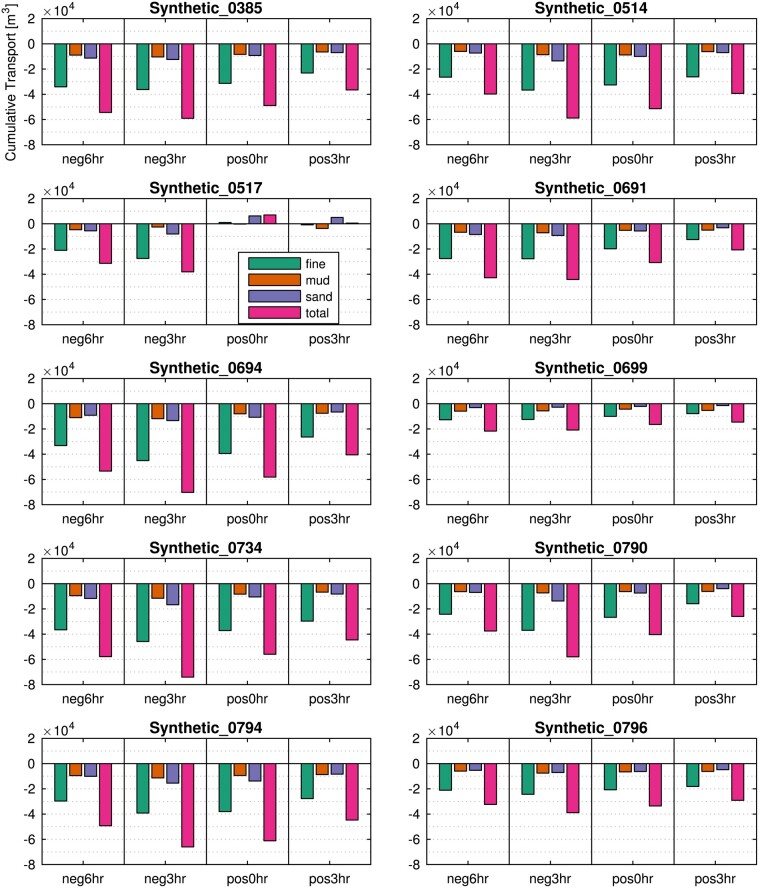
Cumulative sediment flux (as plotted in graph f of Fig. 3) for the different size classes for the 10 synthetic storms and each of the phasing scenarios; sediment flux import is negative, while sediment flux export is positive. Note that except for storm 0517, all grain fractions for all storms and all phasing scenarios show that sediment is imported. The single storm (0517) that does not adhere to this trend exhibits minor exports for only two of the phasing scenarios. These results emphasize that 38 out of 40 model runs result in sediment import. The figure also demonstrates that very fine sand dominates sediment imports, with fine sand and clay being imported in lesser quantities.

**Fig. 5.**
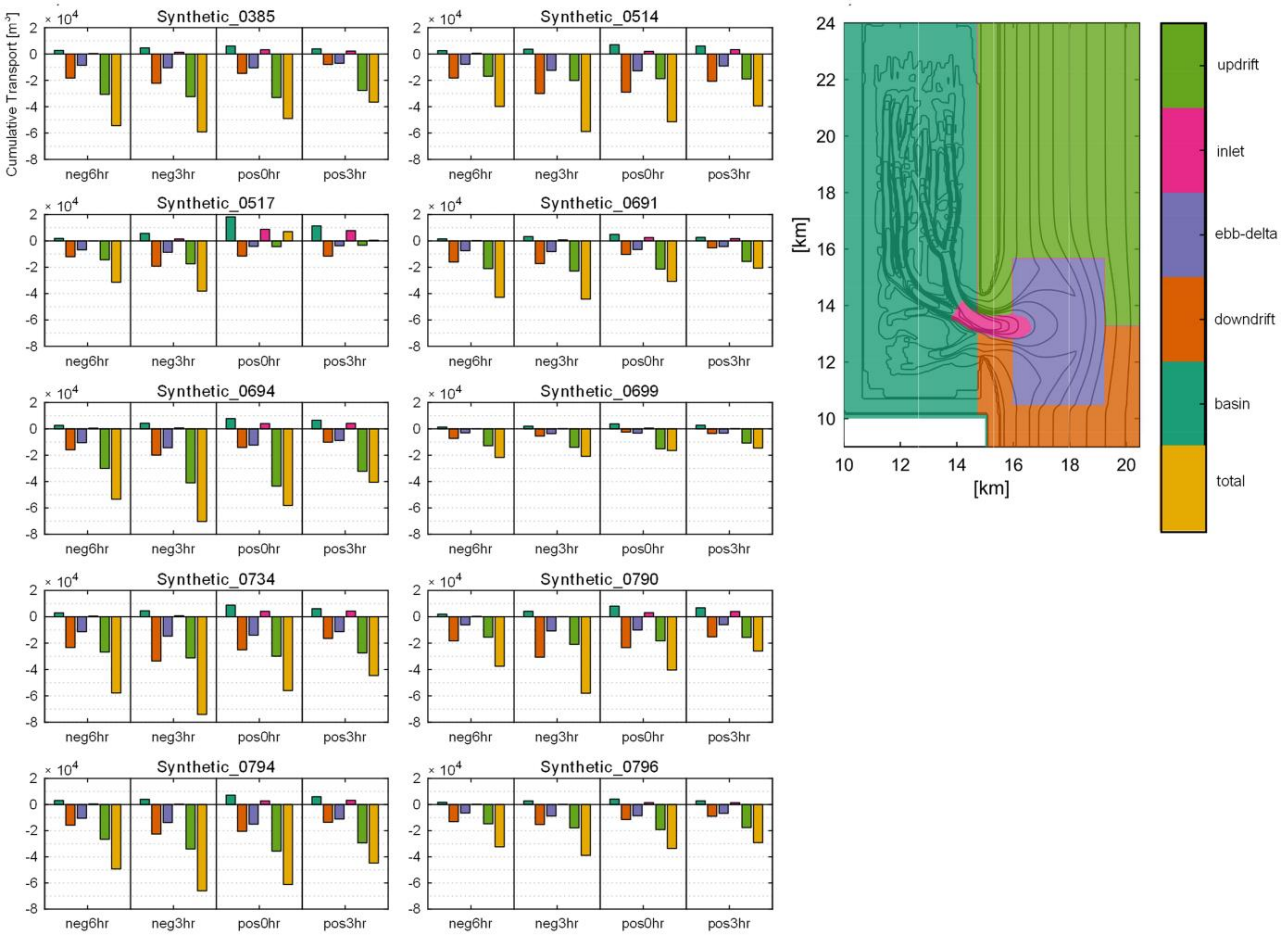
Cumulative sediment flux (as plotted in graph f of Fig. 3) by sediment provenance. Note in the legend that the provenances are represented by: basin; downdrift nearshore; ebb-tidal delta; inlet; updrift nearshore; total cumulative sediment flux. The analyses show that most of the imported sediment is sourced from the updrift nearshore and, secondly, from the ebb-tidal delta and the downdrift nearshore. As expected, exported sediment comes from the basin and tidal inlet, but comparatively these are small volumes. Only storm 0517 shows a net cumulative export for the coincident peak storm surge and high tide scenario (pos0hr).

The cumulative volumetric transport (net sediment flux) for each storm and surge/tide phasing is plotted by sediment type (Fig. 4) and provenance zone (Fig. 5). For all except one of the simulated storms and surge/tide phasing scenarios (storm 0517), experiments import sediment. Where the surge peak leads the high tide by 3 h (neg3hr), the basin experiences the highest sediment import for each storm. When the high tide precedes the surge peak by 3 h (pos3hr), the basin experiences the lowest sediment import. The only experiment with net sediment export (storm 0517) occurs specifically when the surge is in-phase (pos0hr) and lagging the tide (neg3hr).

The timing of the surge peak and high tide influences net sediment transport to the same degree as the characteristics of the storm. Generally, very fine sand contributes the most to sediment import, while the only two experiments that show sediment export are dominated by fine sand (Fig. 4). When inspecting net sediment flux by source (provenance zone) both basin and inlet-sourced sediments are exported, whereas updrift, downdrift, and ebb-delta sediments are imported (Fig. 5). Updrift sediment forms the largest portion of imported sediment, followed by downdrift and ebb-delta sources.

### Influence of storm characteristics

**Fig. 6.**
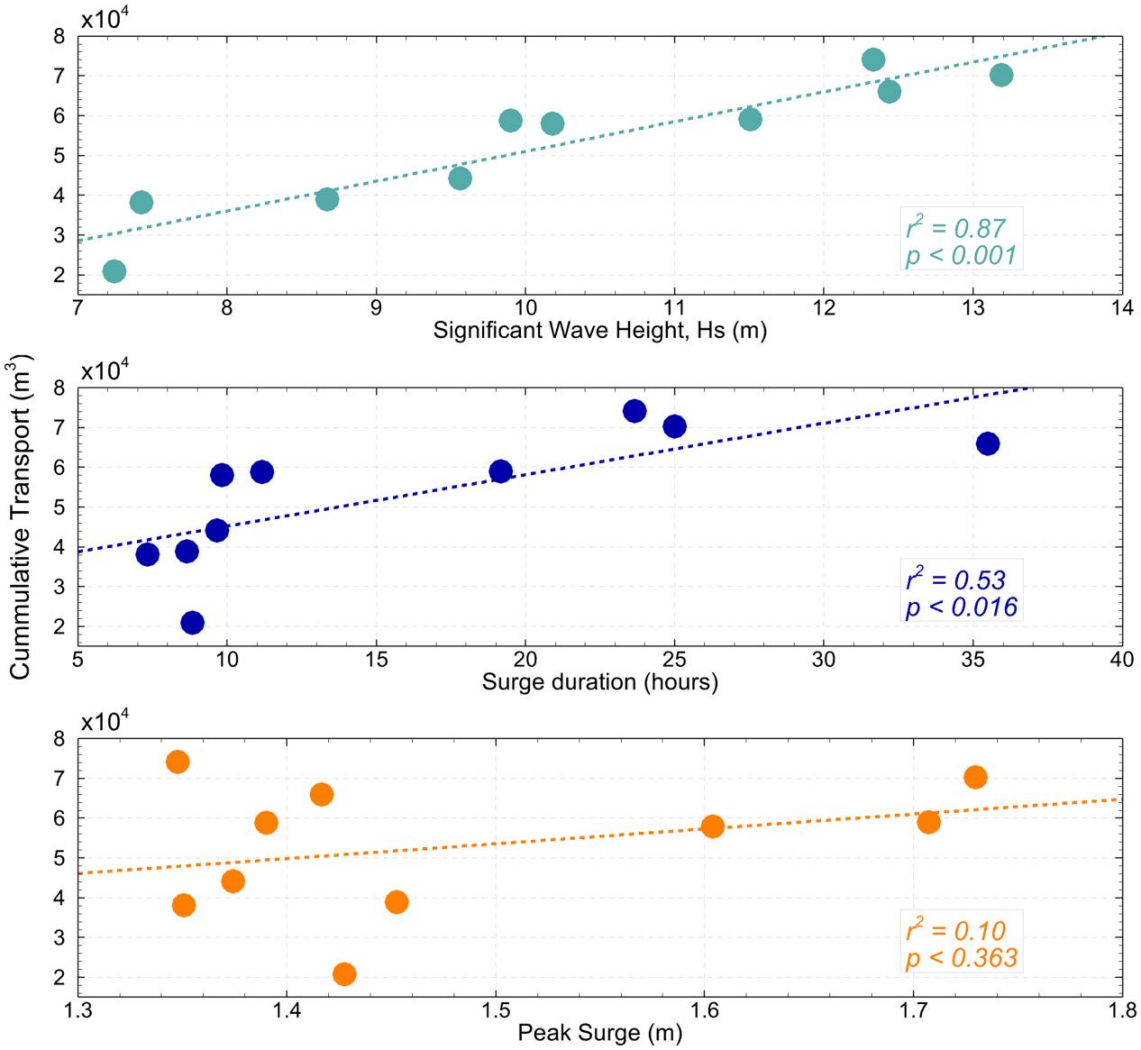
Cumulative transport for the neg3hr scenario plotted against different characteristics of the storm. As seen by the *r*^2^ values, the net cumulative import of sediment correlates best with peak significant wave height (Hs), and secondarily with the duration in which the surge exceeds 0.25 m. Peak storm surge essentially shows no correlation. Peak significant wave height influences bottom shear stresses and sediment suspension, and surge duration influences the period of landward-directed flow.

To investigate the influence of storm characteristics on sediment import and export through the tidal inlet, cumulative transport (Fig. 5) for the neg3hr scenario was evaluated and compared against key storm characteristics ranked in descending order (Fig. 6), including peak significant wave height, storm duration—evaluated when surge exceeds 0.25 m—and peak storm surge (see Figs. S2–S4 for details). Generally, more intense storms import greater volumes of sediment (Fig. 6), consistent with previous findings (25). However, the magnitude of the import shows little correlation with the peak storm surge value. Instead, there are stronger correlations with significant wave height occurring at the peak of the storm surge, and the duration of the storm (Fig. 6).

### Sediment transport, erosion and sedimentation, and sediment provenance

We contrast the processes and mechanisms associated with sediment flux for two storms that have substantively different pathways (alongshore vs. onshore, Fig. 2) and produced different trends. Storm 0385, tracking onshore, generated net sediment import similar in volume to most other simulated storms; the relative magnitude of the import for each experiment (surge/tide phasing scenarios) was also consistent with the other storms; hence, this storm is representative of intense storms that import sediment. Conversely, storm 0517 was the only storm that exported sediment for any surge/tide phasing, and thus, this storm was examined further to determine the specific conditions and mechanisms driving sediment export.

#### Storm 0385 (net import)

Detailed erosional-depositional patterns and morphologic impacts from storm 0385, as well as residual transport for each of the four surge/tide phasing scenarios, are provided (Figs. S5 and S6). For all scenarios, the storm erodes the barrier shoreface, ebb-delta, and inlet margins seaward of the barriers while it deposits sediment in backbarrier channels and inlet margins (Fig. 5). Portions of the flood delta most exposed to increased flood currents and storm wave propagation through the inlet are slightly eroded, while more protected tidal flats experience slight aggradation (Fig. S5). Erosion and sedimentation patterns at the inlet and ebb-delta are notably similar for all scenarios, although when the tide lags surge peak by 6 h (neg6hr) more backbarrier erosion and inlet sedimentation occur compared with other scenarios (Fig. S5). With the lowest water levels during surge peak, the large waves that accompany storm passage have more impact in the backbarrier when flats are not fully submerged.

Residual sediment transport patterns show that with waves approaching primarily from the northeast (storm 0358, 59°, Table 1), net transport in the foreshore is southward, with the highest transport magnitudes along the southern margin of the ebb-delta (Fig. S6). High basinward residual transport occurs in flood channels along the margins of the barriers, while much lower magnitude, net ebb-directed transport occurs at the inlet and through the main channel of the ebb-delta. Most of the sediment moving into the basin is sourced from the updrift, downdrift, and ebb-delta regions and transported through the inlet by flood-directed tidal currents, enhanced by the head caused by storm surge and large waves (Fig. 5). While waves remain high during the subsequent ebb-directed surge outflow, the imported sediment is not transported back out of the basin in significant quantities (Figs. S7 and S8).

**Table 1.** Storm characteristics for the simulated storms.

Storm ID	Track (Fig. 2)	Storm surge peak (m, MSL)	Wave height (Hs, m)	Wave period (Tp, s)	Wave direction (°)	Wind speed (m/s)	Wind direction (°)	Storm size (radius to maximum winds, km)	Forward speed (km/h)
0694	8	1.73	13.2	16.3	52	30.6	78	53	48
0385	1	1.71	11.5	14.9	59	31.6	81	66	60
0790	6	1.60	10.2	12.3	75	35.3	121	76	66
0796	7	1.45	8.7	11.2	64	25.3	97	79	65
0699	4	1.43	7.2	13.5	20	26.6	333	50	60
0794	6	1.42	12.4	14.9	71	37.7	84	49	37
0514	2	1.39	9.9	12.3	79	35.1	127	76	56
0691	3	1.37	9.6	12.3	57	30.2	95	75	66
0517	2	1.35	7.4	9.2	89	37.9	162	52	88
0734	5	1.35	12.3	14.9	73	36.1	91	51	49

The track path is shown in Fig. 2. Other characteristics include the storm surge peak, significant wave height (Hs), peak wave period (Tp), wave direction, wind speed and direction, and the size (Radius to maximum winds) and speed of the storm.

#### Storm 0517 (partial export)

This is the only storm in this study with a pathway parallel to the coast (Fig. 2). Despite having the strongest wind velocities of the group, the storm also exhibited the smallest storm surge and the second-smallest deepwater significant wave height (Hs). Most importantly, it was the only synthetic storm with a net export of sediment from the basin for two of the scenarios (see Fig. 5 and Table 1). For all phasing scenarios, total sediment flux is first directed basinward (import) then seaward (export) coincident with storm impact. However, the relative magnitude of the sediment imports and exports preceding and following the storm surge peak influences the net flux direction over the full simulation (see Fig. S9).

Notable for this storm is that the neg6hr and neg3hr surge/tide phasing scenarios produce a net sediment import, whereas the pos0hr phasing produces no import. The pos3hr phasing, where the surge peak follows high tide by 3 h, produces sediment export (Fig. S9). For the pos3hr phasing, the flood-directed current speeds preceding the surge peak are the lowest of all phasing scenarios, and the subsequent ebb-directed outflow surge currents are the greatest. Additionally, wave heights significantly decrease during the sudden lowering of water levels after surge peak, which could decrease the import of coarser sediments at the inlet margins as evidenced by erosion/deposition patterns and residual transport (Figs. S10 and S11).

## Discussion

Our results highlight storm impacts on backbarrier systems at mixed-energy barrier/tidal inlet systems (see Table S1 for similar worldwide locations), where storm surges during intense storms are similar in magnitude to tidal amplitudes. The foremost finding from the 40 scenarios we modeled is that storms generate a net import of sediment through the inlet and into the tidal basin, and most of this sediment is sand sourced from the updrift and downdrift nearshore adjacent to the inlet as well as from the ebb-tidal delta (Fig. 5). Our analyses consistently show that when surge peak leads high tide by 3 h, the greatest volume of sediment is imported through the inlet (neg3hr, Fig. 5). During this scenario, fine and very fine sands are transported into the basin (Fig. 4) coincident with enhanced flood currents focused in the marginal-flood channels (shallow channels located slightly seaward and adjacent to the inlet). Even for surge/tide phasing scenarios that favor high ebb-directed surge outflow currents (pos0hr and pos3hr), the wave-enhanced flood currents in the shallower inlet margins produce higher sediment import than the subsequent ebb-directed currents that are more confined to the inlet throat, a mechanism that explains sediment import for most storms across all phasing scenarios (Fig. 5). Very fine sand is mobilized and transported basinward to a greater degree than fine sand such that it dominates the residual import for all storms (Fig. 4). Moreover, offshore clay remains in suspension after initial import due to low settling velocities (9) and is subsequently exported, resulting in a lower import of clay than sand. Still, some fine sediment settles across the backbarrier, consistent with field observations of post-storm deposition (35–37).

Along the Virginia coast, storms were shown to import mud through the tidal inlets (25), but conversely, on the basin side of tidal inlets, fine sand was eroded, while on the ocean side of inlets, very fine sand was deposited. Differences in outcomes of these studies might be explained by the lower tidal range of the Virginia coast when compared with the conceptualized tidal range in this study, causing the influence of the storm surge along the Virginia coast to dominate. Field studies and numeric modeling at site-specific locations corroborate that sediment is imported to backbarriers through some tidal inlets. One of these studies based the conclusion on a net sediment deficit, whereby only a marine source of sediment would balance the sediment budget (Plum Island Sound, MA (38)). A modeling study of Rockaway Inlet and Jamaica Bay along Long Island, New York showed marine sediment is imported into the Bay and that storms may enhance the influx (20). The hydrodynamics at Rockaway Inlet–Jamaica Bay during Hurricane Sandy were simulated using a Delft3D modeling platform and calibrated with field measurements (39). They reported that 9,400 metric tons entered the bay during the single event, with mud (53%) constituting a slightly greater fraction than sand (47%). In addition, a field study at New Inlet in Massachusetts using hydrographic and suspended sediment data argues that much of the sediment deposited on the estuarine marsh is storm derived from the marine environment (40).

A single storm with a track parallel to the coast (storm 0517) produces conditions in which two of the phasing scenarios (pos0hr and pos3hr) caused a relatively small net export of sediment out of the basin. This export of sediment contrasts sharply with the other storm scenarios and can be explained by the shore-parallel (and proximal) storm track that produces a rapid shift in wind directions from onshore to offshore and a steep drop in water level as the northward-tracking storm passes the basin latitude. The high ebb-directed surge outflow current superimposed with the ebb-tidal current in these two phasing scenarios creates ebb-dominant residual currents and small net sediment export.

Unlike microtidal environments, where surges and surge-induced currents through inlets substantially exceed those during calm periods (41), the relative surge/tide phasing at mesotidal inlets becomes a significant factor controlling the hydrodynamics and residual transport within the system. While the relative phasing of surges and tides has been investigated for impacts on nonlinear surge dynamics (42), phasing impacts on residual sediment transport into or out of basins due to storms are relatively unexplored (6, 25). Our results show that in addition to the relative surge/tide phasing, sediment transport into the basin is also affected by the characteristics of the storm (Fig. 6). We find that cumulative transport for the neg3hr scenario correlates best with peak significant wave height and, secondly, with the duration in which the storm surge exceeds 0.25 cm above the predicted high tide. Little correspondence was found with peak storm surge. Peak significant wave height is an overriding factor in dictating bottom shear stresses and sediment suspension on the ebb-tidal delta and the nearshore, while duration of the storm surge affects the duration of the flood-directed currents. Both these parameters are significant in determining the magnitude of sand delivered to the inlet and generating landward sand transport to the backbarrier.

When surge peak and high tide are in phase (pos0hr), flood-tidal currents and surge inflow coincide to the greatest degree; however, this phasing produces slightly lower import. The highest influx of sand for each storm occurs when the surge peak leads high tide by 3 h (neg3hr) coinciding with the strongest flood currents slightly preceding both surge peak and mid-tide. This occurs when the superimposed water levels are <2 m (Fig. 3), and influence of waves on the bottom are enhanced. The high ebb-currents developed during the falling tide export some sediment, which reduces the storm cumulative import. For both scenarios (pos0hr and neg3hr), high waves persist during the passage of the storm without substantial differences in wave impacts during the flood and ebb periods; however, the high waves entrain coarse sediments more readily at the shallower inlet margins where flood currents dominate, creating a residual flood transport across the inlet (see Fig. S6). Also, we acknowledge that phasing of the storm with respect to spring vs. neap tidal conditions maybe an important factor, especially in areas with larger tidal ranges because of greater differences in neap vs. spring tidal elevations.

Simulated storm impacts on ebb-tidal delta morphology are generally as expected (35). Sediment of all sizes is eroded throughout the ebb-tidal delta, with the greatest erosion focused on the terminal lobe. While Miner et al. (35) observed erosion of the ebb-tidal delta at Little Pass Timbalier, potentially providing a sediment source for widespread backbarrier marsh sedimentation (35), this research finds that updrift and downdrift sources contribute relatively more sediment for backbarrier deposition, though the boundaries between the barrier shoreface and marginal-flood channels denoting updrift/downdrift or ebb-delta provenance are imprecise. Fine sand and very fine sand are still cumulatively most important in the sediment influx, indicating that the results are generally applicable to sandy basins with similar morphology, hydrodynamic, and storm conditions (27). This conceptual model lacks the effects of marsh vegetation on both hydrodynamics and sediment transport on backbarrier marsh platforms. However, the transport of coarse sediment (sand) through the inlet-associated marginal-flood channels and from the ebb-delta to the flood-tidal delta and proximal backbarrier tidal creeks would be unaffected by marsh. Moreover, cohesive sediments in marsh-dominated estuaries and lagoons are still mostly deposited on tidal flats and in shallow channels (43).

Our results explain the formation of flood-tidal deltas (44) and development of other sand bodies inside inlets in backbarrier basins. Conceptually, during storms large wave heights produce greater than normal shear stresses that suspend increased quantities of sand off the bottom in the nearshore and on the ebb-delta. The storm surge coupled with the rising tide produces a steep water surface slope into the inlet and strong flood currents, which carry the sand into backbarrier. Once beyond the confines of the inlet throat, channels bifurcate, widen, and collectively expand in a cross-sectional area, causing the currents to slacken and sand to be deposited.

Similar tidal basins are expected to import more sediment with future increases in storminess (29, 45) and SLR, altering the long-term sediment balance of the coastal cell comprising the inlet, ebb-delta, and adjacent barrier shorelines (14, 21, 46–50). While a long-term shift to basin import accentuated by episodic, storm-induced import will increase the available sediment for redistribution within the basin and potentially enhance salt marsh accretion, it will be at the expense of the nearshore sediment supply that could hasten barrier narrowing, transgression, and ultimately breaching (21). In summary, our process-based numerical model of a conceptual basin-tidal inlet system demonstrates the impact of major storms on residual sediment fluxes, suggesting that tidal inlets will be important long-term sediment sinks, impacting the management of adjacent shorelines. Limited sensitivity analyses on the influence of tidal range and clay content in the storm modeling showed only minor differences in the magnitude and provenance and no changes in the net import or export of sediment (see Supplementary material).

## Materials and methods

### Model setup and design

The numerical model used in the study included relevant morphologic features of a barrier/tidal-inlet-basin system and included the processes that drive bed level changes during storms and sediment exchange between the basin and environments seaward of the inlet. Storm impacts on basin hydrodynamics, sediment transport, and exchange between morphologic elements are investigated with an idealized basin model so that relationships and storm responses can be more easily quantified and compared. The model hydrodynamic and morphology grids were adopted from a previous study (22) and consist of an elongated basin of ∼15 km by 5 km (with 50 m resolution) and a 30-km alongshore by 10-km cross-shore section of the nearshore, nested within a 60-km by 15-km coarse-resolution (500 m) wave grid (Fig. 1). Hydrodynamic and sediment-transport simulations were conducted using the fluid dynamic model Delft3D-FLOW (51) and the Delft3D-WAVE module, which is based on the SWAN (Simulative WAves Nearshore) wind wave generation and propagation model (52). The Delft3D modeling suite is ideal for simulating unsteady flows, wave generation, propagation, and transformation in shallow water, sediment transport, and bed level changes due to transport gradients of cohesive and noncohesive sediments, and morphological up-scaling techniques that extend the practical time horizons of simulations (51–55).

The conceptual inlet-basin system Delft3D hydrodynamic, wave, sediment transport, and morphologic model domain and initial bathymetry are shown in Fig. 1. The initial bathymetry represents an approximate equilibrium condition reached after a 1.5-m amplitude semi-diurnal, sinusoidal tide along with a 0.25-m significant wave height, 6-s peak period wave at 75° N was imposed for several years of simulation time with computed sediment fluxes to and from the bed scaled up by a factor of 50 (Delft3D MORFAC feature—see (53)). Using an undistorted tidal signal imposed at the model boundaries, any asymmetries developing during the modeling are caused by variations in the inlet and basin geometry with water level (9, 56). Both the basin dimensions and tidal environment approximate the characteristics of typical mixed-energy, mesotidal systems along the US New England coast (27), such as Plum Island Sound (Fig. 1a). Basin extents are fixed, mimicking the anthropogenic infrastructure and other geologic controls that limit upland migration of many coastal wetlands (8). The initial model bathymetry and tidal hydrodynamics produced by the long-term simulation with stationary, constant wave-climate boundary conditions are broadly consistent with mixed-energy, tide-dominated basins (57) with well-developed ebb-delta and flood-tidal delta (58) and ebb-dominant tidal asymmetries that export sediment under typical tidal conditions (27).

To ensure that the surge peak timing during the spring or neap tidal conditions did not influence the model results and outcomes, a sensitivity analysis was adopted and included evaluating lower and higher tidal amplitudes. Two additional tidal amplitudes, one lower (1 m) and one higher (2 m), were selected, and we simulated storm 0385 for these two tidal conditions, and all four phasing scenarios for a total of eight additional simulations. In addition, because finer sediments such as mud are important to the resilience of backbarrier marshes, additional sensitivity experiments were conducted by changing clay content. Two additional scenarios were simulated, with the clay content reduced from 14 to 4%, and also increased from 14 to 24%. Again, storm 0385 was repeated for these two clay content conditions for all four tidal phasing scenarios, yielding eight more simulations (see Supplementary material, Figs. S18 and S19).

### Synthetic storm selection

To model the effects of tropical storms on mixed-energy barrier/inlet systems in New England, storm surge, wind, and wave boundary conditions associated with the storm are required. As an alternative to historic storms impacting the region, where complete measurements of concurrent wind, wave, and water level data may be unavailable, synthetic storms simulated as part of the United States Army Corps of Engineers (USACE) North Atlantic Coast Comprehensive Study (NACCS) (33) were used in this study. Synthetic storms used in our study were selected to be representative of the hurricane climatology following the Joint Probability Method with Optimum Sampling methodology (59, 60). From the suite of available storms, a subset of 10 storms was selected, and output from those 10 storms was extracted at a location ∼20 km offshore of Plum Island Sound (see Fig. 2). The storms were sorted by maximum surge elevation, and then the 10 highest storm surges were selected based on the hypothesis that more intense storms (as measured by surge magnitude) would produce greater impacts on the sediment dynamics of the system. The characteristics of the selected storms are given in Table 1 and their storm tracks are shown in Fig. 2.

### Boundary condition development

Extracted water levels for NACCS synthetic storms were derived from the base-case simulations, where no tidal forcing was applied. To develop boundary conditions that include tides, surge levels were superimposed with a 1.5-m amplitude sinusoidal tide, approximately equal to the mean tidal amplitude along the northern Massachusetts and Merrimack Embayment Barrier Chain coastlines (27). For each storm, four surge/tide phasing scenarios were developed where high tide leads the surge peak by 6 h (out of phase), 3 h, 0 h (in phase), and lags by 3 h—scenarios referred to as “neg6hr,” “neg3hr,” “pos0hr,” and “pos3hr,” respectively. This type of superposition represents a simplified approach to the water level boundary conditions, as it neglects potential nonlinearities in tidal and surge interactions (42). With four surge/tide phasing scenarios for each storm, a total of 40 hydrodynamic, sediment transport, and morphology simulations were performed for this study. Each simulation was run for a period of 8 days, with the CHS-extracted boundary conditions shifted such that the peak storm surge occurred at the start of the fifth day of simulation for all storms and surge/tide phasing scenarios.

Significant wave height, peak wave period, mean wave direction, wind speed, and wind direction time series were also extracted for the synthetic storm simulations at the NACCS output location where surge was extracted (Fig. 2). The CHS system (34) only provides time series output for the above parameters for the duration of the storm peak, typically 1 to 2 days. Thus, the time series for each parameter were extended for several days before and after the storm peak for use in the Delft3D simulations to better evaluate tidal and storm interaction. For the wave parameters, the significant wave height during prestorm and poststorm periods was assumed to be ∼0.25 m to represent background wave energy, while the peak wave periods and mean wave directions for periods with no available data were extrapolated with the nearest-neighbor peak period. Finally, winds were assumed to be calm prestorm and poststorm and were thus reduced to near zero value. An example of the derived boundary conditions for the Synthetic_0385 storm simulation with neg3hr surge/tide phasing is provided in Fig. S1.

### Sediment setup and source tracking

The sediment bed at the start of the simulation is composed of a 3-m-thick, horizontally and vertically uniform mixture of three sediment classes with the corresponding median grain diameters: 200 μm (fine sand), 100 μm (very fine sand), and cohesive clay with a fall velocity of 2.5e−4 m/s (corresponding to an approximate median grain diameter of 20 μm assuming Stokes’ settling (54)). Based on the maximum expected erosion observed in test runs, the 3-m-thick bed of uniformly mixed sediment represents an unlimited bed supply condition where each fraction comprising the bed is available for transport in proportion to its volume fraction when eroded (61). The fine and very fine sand fractions each make up ∼43% of the bed, whereas the clay fraction comprises ∼14% of the bed by volume. While the bed is initially defined as vertically uniform, the upper portion of the bed (active transport layer) is divided into 20 layers of 5 cm thickness with compositions that are updated as erosion or deposition of specific sediment fractions occur. Each of the three size fractions is then further divided spatially into five regions based on the initial location within the model domain—denoting provenance from the updrift, inlet, ebb-delta, downdrift, or basin zones of the model domain. Thus, the provenance of sediment transported through the inlet can be ascertained in a manner similar to previous studies (62). Given 3 size classes and 5 provenance zones, a total of 15 sediment fractions are simulated during each simulation.

## Supplementary Material

pgae042_Supplementary_Data

## Data Availability

All data to reproduce the analysis in this manuscript are included as attachments to the reviewer assessment, including model files. All model simulation setup files are also included and can be found in the following data repository: https://zenodo.org/doi/10.5281/zenodo.10558887. The model setup files include the initial conditions, delineation of the sediment provenance zones, and all the storms that were simulated, and include cumulative sediment fluxes at the inlet cross-section used in the analysis of the study. The numerical model used in the analysis is the Delft3D-4 modeling suite and is available in the public domain. The software and computer source code are available at https://oss.deltares.nl/web/delft3d/downloads.
