# Supplementary material for: Storm and tidal interactions control sediment exchange in mixed-energy coastal systems
Source: PNAS Nexus. 2024 Feb 3;3(2):pgae042. doi: 10.1093/pnasnexus/pgae042 (PMC10898511; doi:10.1093/pnasnexus/pgae042)
Supplement: pgae042_Supplementary_Data [file pgae042_supplementary_data.zip › PNASNEXUS-PNASNEXUS-2023-00832R-s03.docx]

**Supporting Material for**

Storm and tidal interactions control sediment exchange at mixed-energy coastal systems

Ioannis Y. Georgiou^1,*^, Duncan M. FitzGerald^2^, Kevin Hanegan^3^

^1^ The Water Institute, 2021 Lakeshore Dr., Suite 310, New Orleans LA 70122

^2^ Boston University, Department of Earth and Environment, 685 Commonwealth Avenue, Boston MA 02215

^3^ Moffatt and Nichol, 601 Poydras St, New Orleans LA 70130

* Ioannis Y. Georgiou. 2021 Lakeshore Dr., Suite 310, New Orleans, LA 70122, ++504-931-5178

**Email:**  [igeorgiou@thewaterinstitute.org](mailto:igeorgiou@thewaterinstitute.org)

**This file includes:**

S1. Hydrodynamic and Sediment Transport Model Development and Validation

Figures S1 - S19

Table S1

SI References

**S1. Hydrodynamic and Sediment Transport Model Development and Validation**

**Introduction.** To ensure model skill, despite simulating an idealized basin, we validated the sediment transport module using a representative mixed-energy tidal inlet and basin system. The objective was two-fold. First, to ensure that the assumptions of sand and mud contained within the sediment bed is reasonable, and second, that the selection of sediment constants for erosion, ie, critical shear stress from erosion, reproduced observations in a system with similar energy reasonably well. We selected the validation to be in Plum Island Sound, MA, because the schematized model was informed by this system, and because suspended sediment concentration (SSC) observations (seasonal) are available from previous efforts. A previous developed hydrodynamic (Delft3D) model ^1^ used to simulate hydrodynamic conditions during an intense extratropical cyclone in January 2018 that influenced Plum Island Sound.

**Model Development.** The hydrodynamic and morphologic grid covers a rectilinear area with dimensions of approximately 20 km by 13 km that includes Plum Island Sound, the connecting Parker River tidal inlet, the most downstream reach of the Merrimack River, and a portion of the Gulf of Maine extending offshore to depths exceeding 20 m ^2^. The resolution of the hydrodynamic grid is 20 m at the inlet and within PIS, with reduced resolution in offshore areas. The computation grid for wave calculations is identical to the hydrodynamic grid but with resolution reduced by a factor of three in the back‐barrier basin. Model bathymetry was developed from hydrographic survey data collected in 2011 and 2015 and LiDAR topographic elevations collected in 2011 and 2014 ^2^. Figure S13 displays the domain, offshore boundary locations, and bathymetry for the PIS Delft3D model. The flow from rivers discharging into PIS was schematized with point sources providing constant discharges equal to the monthly average discharges ^3^. With this study’s focus on the internal redistribution of sediments within the system due to storms, the minor ^4^ suspended sediments provided by these riverine sources were neglected.

**Model Validation.** The PIS Delft3D hydrodynamic model was previously calibrated for simulated water levels and tidal astronomic constituents within PIS as well as for the tidal prism at Parker River Inlet ^1,2^. For this work extending the model for sediment transport, the ability of the model to simulate typical backbarrier Suspended Sediment Concentrations (SSC) was verified. Seasonal measurements of total SSC in various areas and tributaries of PIS were available from recent (2016 and 2017) measurement campaigns ^5^. In Figure S15, the spatially‐averaged total SSC from each seasonal sampling event are summarized, where total mean SSC during fall and winter greatly exceeds that of summer, and seasonal averages vary between approximately 1 and 20 mg/L, consistent with other long‐term sampling of PIS SSC^6,7^. To verify model SSC predictions, a month‐long simulation with only tidal forcings ^8^ was conducted for comparison to measurements. In Figure S16, SSC time series at a representative output point in a PIS tidal channel (see Figure S13 for the location of the PIS1 output point) shows that the total SSC varies strongly with the spring‐neap tidal cycle – confirming that tidal currents are the primary process responsible for sediment resuspension and dispersal in PIS ^4,7^. The simulated total SSC is mainly composed of clays and very fine sands, with much lower concentrations of medium sand, and the time‐averaged total SSC across the simulation period is on the order of 1 mg/L, consistent with summer measured averages when stirring of sediments due to waves is minimal. Figure S17 plots the spatial variation in time‐averaged per size fraction and total SSC; fine sand SSC is the highest over shallow flats and portions of the ebb‐delta where flow velocities are high, while clay SSC concentrations only show minor variation within the proximal flats, primary tidal channels, inlet, and ebb‐delta of PIS. Table S1 gives the values of cohesive fractions critical shear stress for erosion and the Van Rijn “Sus” and “Bed” calibration factors used to produce simulated backbarrier SSC values consistent with measurements.

A

C

B

**Figure S1.** Plot of boundary conditions for Storm 0385 with negative 3-hour phase scenario in which high tide leads storm surge peak by 3 hours: A. Water level, developed by superimposing a 1.5 m sinusoidal tide with the United States Army Corps of Engineers North Atlantic Coast Comprehensive Study (NACCS) zero moment wave height, and peak wave period (extracted from NACCS STWAVE simulations), B. Corresponding wave and wind direction (° N) from NACCS simulations, and C. Wind speed.

**Figure S2.** Cumulative sediment flux (gold bar), sorted by peak storm surge (measured in meters along horizontal-axis, left to right). Each subplot contains results for all storms with common surge/tide phasing scenario. The synthetic storm ID is shown in small fonts above the horizontal axis. Note that little correspondence exists between cumulative transport and storm surge elevation (see Figure 6).

**Figure S3.** Cumulative sediment flux, sorted by peak significant wave height (horizontal axis) at the boundary. Each subplot contains results for all storms with common surge/tide phasing scenario. Note the correspondence that exists between cumulative sediment flux (gold bar) and peak significant wave height (see Figure 6).

**Figure S4.** Cumulative sediment flux, sorted by the duration in which the surge exceeds 0.25 m (measured in hours, plotted along horizontal axis). Each subplot contains results for all storms with common surge/tide phasing scenario. Note a close correspondence between cumulative sediment flux (gold bar) and surge duration (see Figure 6).

**Figure S5.** Patterns of cumulative sediment erosion and deposition for Storm 0385 for the four tidal phasing scenarios.

**Figure S6.** Residual sediment transport patterns and magnitudes at the tidal inlet, ebb-delta, and flood-delta for synthetic storm 0385 for the four tidal phasing scenarios.

**Figure S7.** Summary of results of Storm 0385 for neg3hr surge/tide phasing scenario showing: a) Simulated water level at inlet resulting from a boundary condition that superimposes the modeled storm surge from the NACCS study with a 1.5 m amplitude sinusoidal tide having a peak surge that leads high tide by 3 hours, b) Simulated significant wave height (Hs), and c) Simulated channel-averaged current speed at the inlet. Plots d-e-f are simulated cumulative sediment flux through the inlet during storm period by total and per provenance zone for d) very fine sand, e) clay, and f) fine sand. The vertical black line in all subplots marks the time of peak storm surge.

**Figure S8.** Cumulative change in per unit area sediment mass for each provenance zone (columns) and size fraction (rows) for Storm 0385 neg3hr surge/tide phasing scenario. Negative values indicate a net erosion and positive values indicate a net deposition of the particular size class and provenance zone sediment.

**Figure S9.** Summary of results for Storm 0517 at inlet for each of the four simulated surge/tide phasing scenarios (neg6hr, neg3hr, pos0hr, pos3hr): a) Water level at inlet superimposing the modeled storm surge from the NACCS study with a 1.5 m amplitude sinusoidal tide for various phases, b) Significant wave height (Hs), c) Total sediment concentration (sum of all grain size fractions), d) Simulated water discharge through the inlet, e) Total (sum of all fractions) sediment flux through the inlet, and f) Cumulative sediment flux through the inlet during storm period, indicating tidal basin import or export of sediment. Storm 0517 produced an import of sediment for the neg6hr and neg3hr surge/tide phasing scenarios and export for pos0hr and pos3hr phasing.

**Figure S10.** Cumulative erosion and deposition for Storm 0517 for the four tidal phasing scenarios.

**Figure S11.** Residual sediment transport vectors at the tidal inlet, ebb-delta, and flood-delta for synthetic storm 0517 for the four tidal phasing scenarios. Note that pos0hr and pos3hr are the only scenarios that produced net cumulative export of sediment.

**Figure S12.** Summary of results of Storm 0517 for pos0hr surge/tide phasing scenario showing: a) Simulated water level at inlet resulting from a boundary condition that superimposes the modeled storm surge from the NACCS study with a 1.5 m amplitude sinusoidal tide having a peak surge that leads high tide by 3 hours, b) Simulated significant wave height (Hs), and c) Simulated channel-averaged current speed at the inlet. Plots d-e-f are simulated cumulative sediment flux through the inlet during storm period by total and per provenance zone for d) very fine sand, e) clay, and f) fine sand. The vertical black line in all subplots marks the time of peak storm surge.


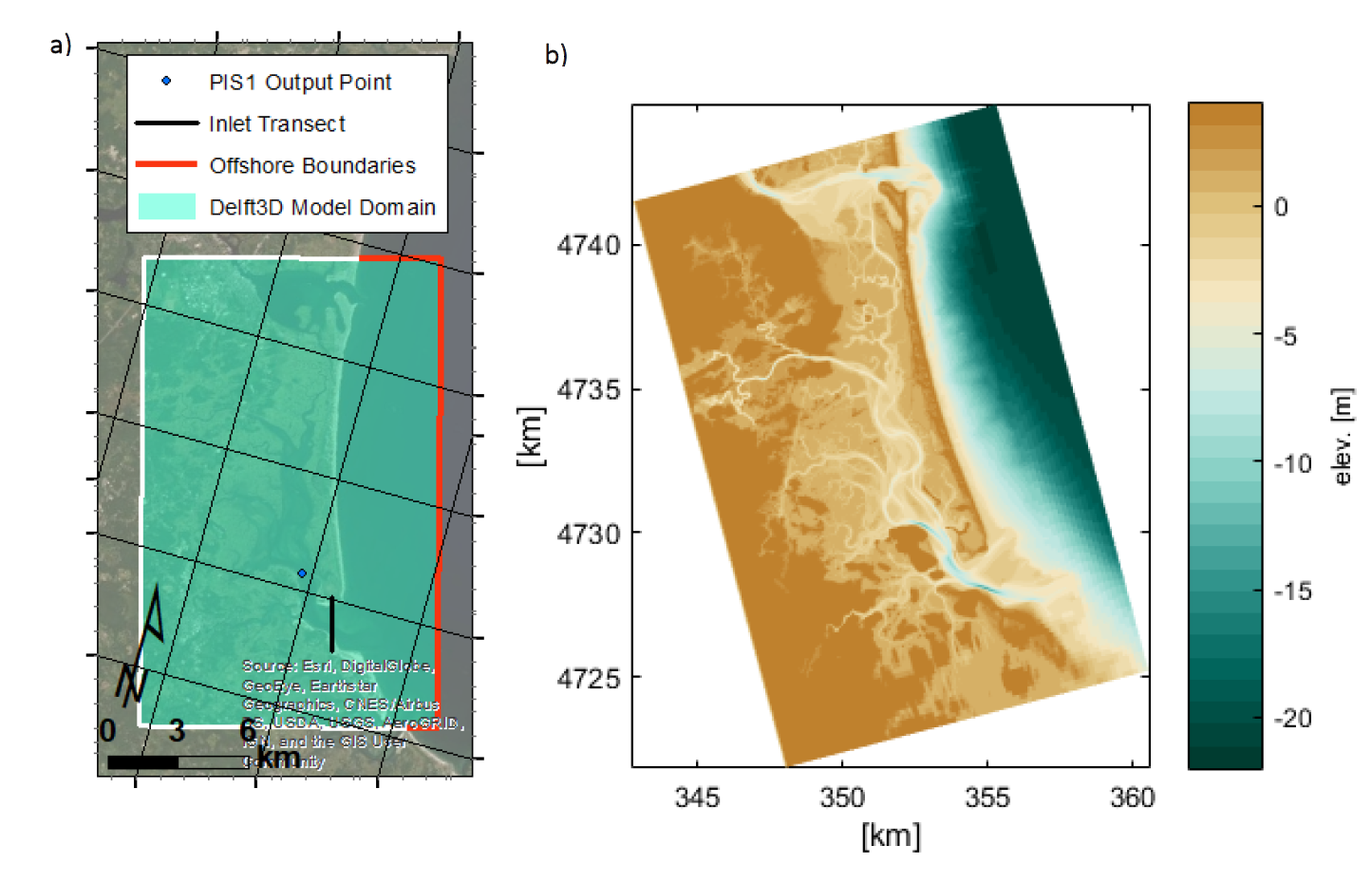


**Figure S13.** Aerial photograph mosaic (a) of Plum Island Sound (PIS) in northern Massachusetts, USA, and Delft3D hydrodynamic, sediment transport, and morphologic model domain showing offshore boundaries, and (b) model initial bathymetry.


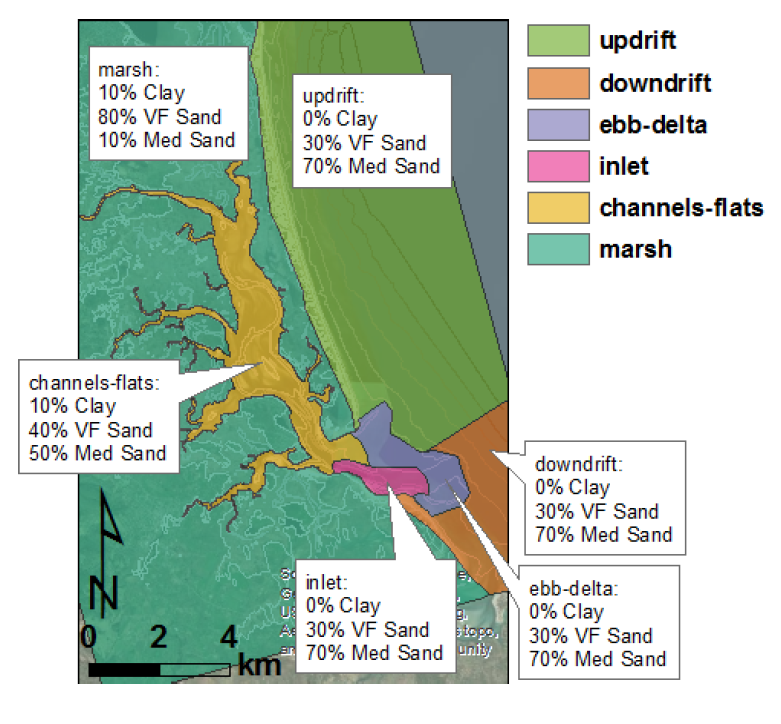


**Figure S14.** Zones used to track the provenance of transported sediment, shown along with initial model bathymetry contours and the initial volume fraction of each size class specified within the bed of each zone. For each size fraction (Clay, Very Fine (VF) Sand, and Medium (Med) Sand), six different sediment classes corresponding to each provenance zone were simulated, enabling tracking of the sediment source for deposition and transport through the inlet. In subsequent plots, the six zones are abbreviated with their first letter: updrift (u), downdrift (d), ebb-delta (e), inlet (i), channels flats (c), and marsh (m).


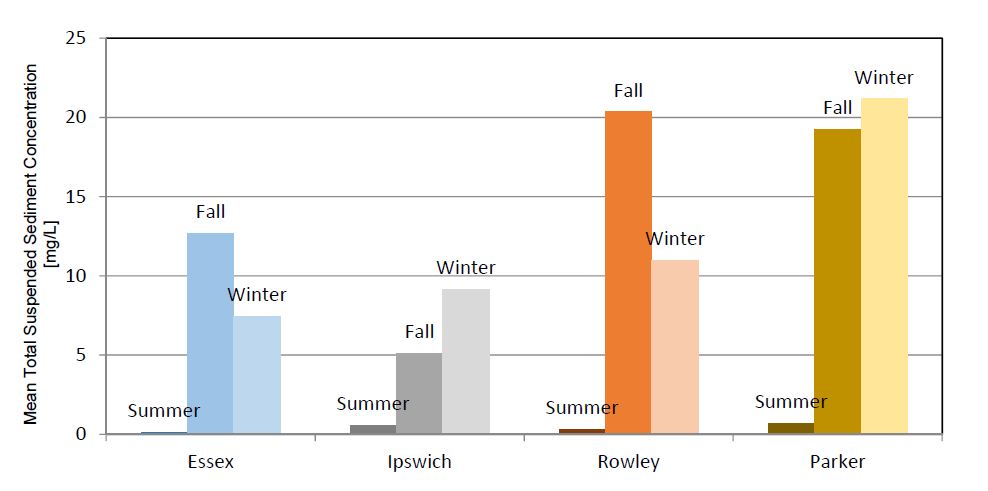


**Figure S15.** Mean (averaged over sampling distance) total suspended sediment concentration in areas of the Plum Island Sound estuary measured during the summer of 2016, fall of 2016 (November 17, 2016), and winter of 2017 (February 24, 2017)^5^.


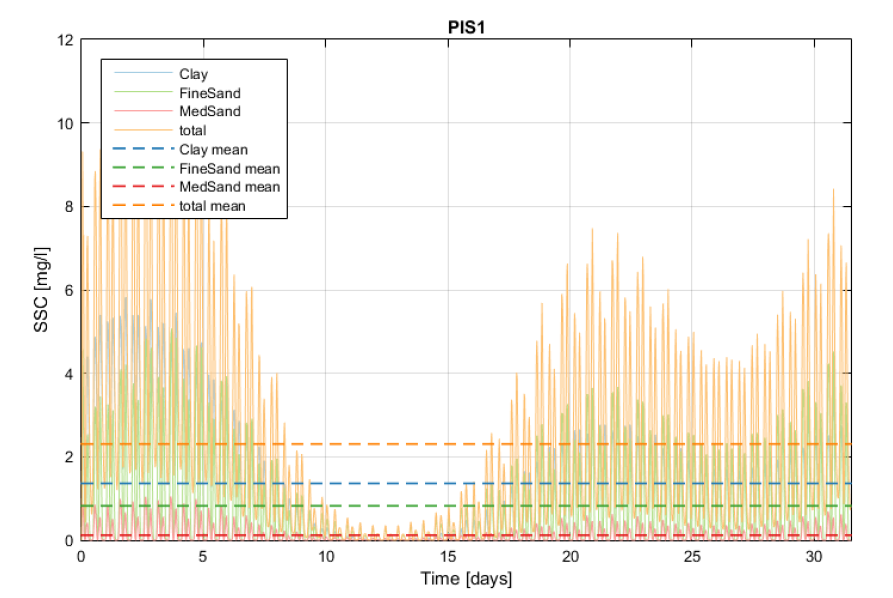


**Figure S16.** Simulated total and per size fraction suspended sediment concentration at a representative output point in the primary backbarrier tidal channel of PIS (see Figure S13 for PIS1 output point location). Total SSC is mostly composed of very fine sand, and time-averaged total SSC is on the order of 10 mg/L, consistent with seasonal measurements ^5^.


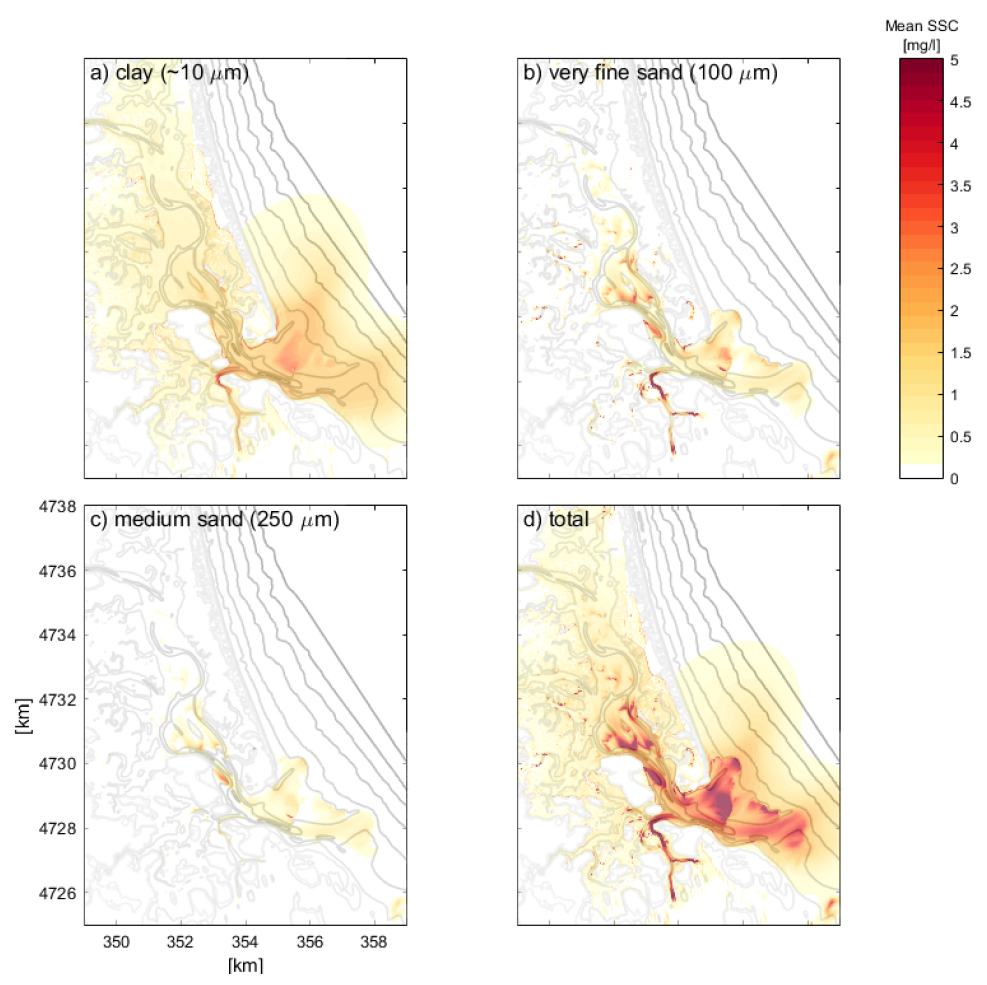


**Figure S17.** Simulated time-averaged suspended sediment concentration (per size fraction (a-c) and total (d)) at PIS for a monthlong simulation using only astronomic forcings. Fine sand SSC is highest over shallow flats and portions of the ebb-delta where flow velocities are high, while clay SSC concentrations only show minor variation within the proximal flats, primary tidal channels,

inlet, and ebb-delta of PIS.

**Table S1.** Characteristics of sediment classes used in Delft3D model, including the most important settings used to verify the simulation of clay (cohesive) and very fine and medium sand (non-cohesive) SSC.


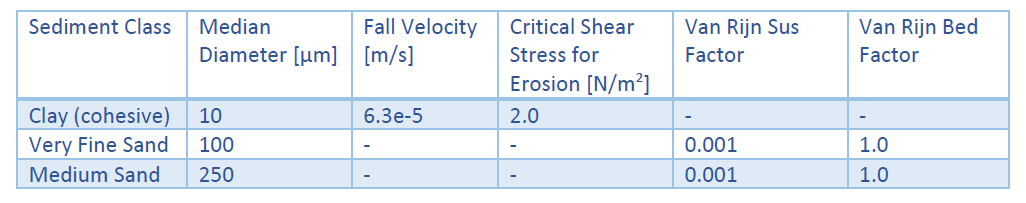


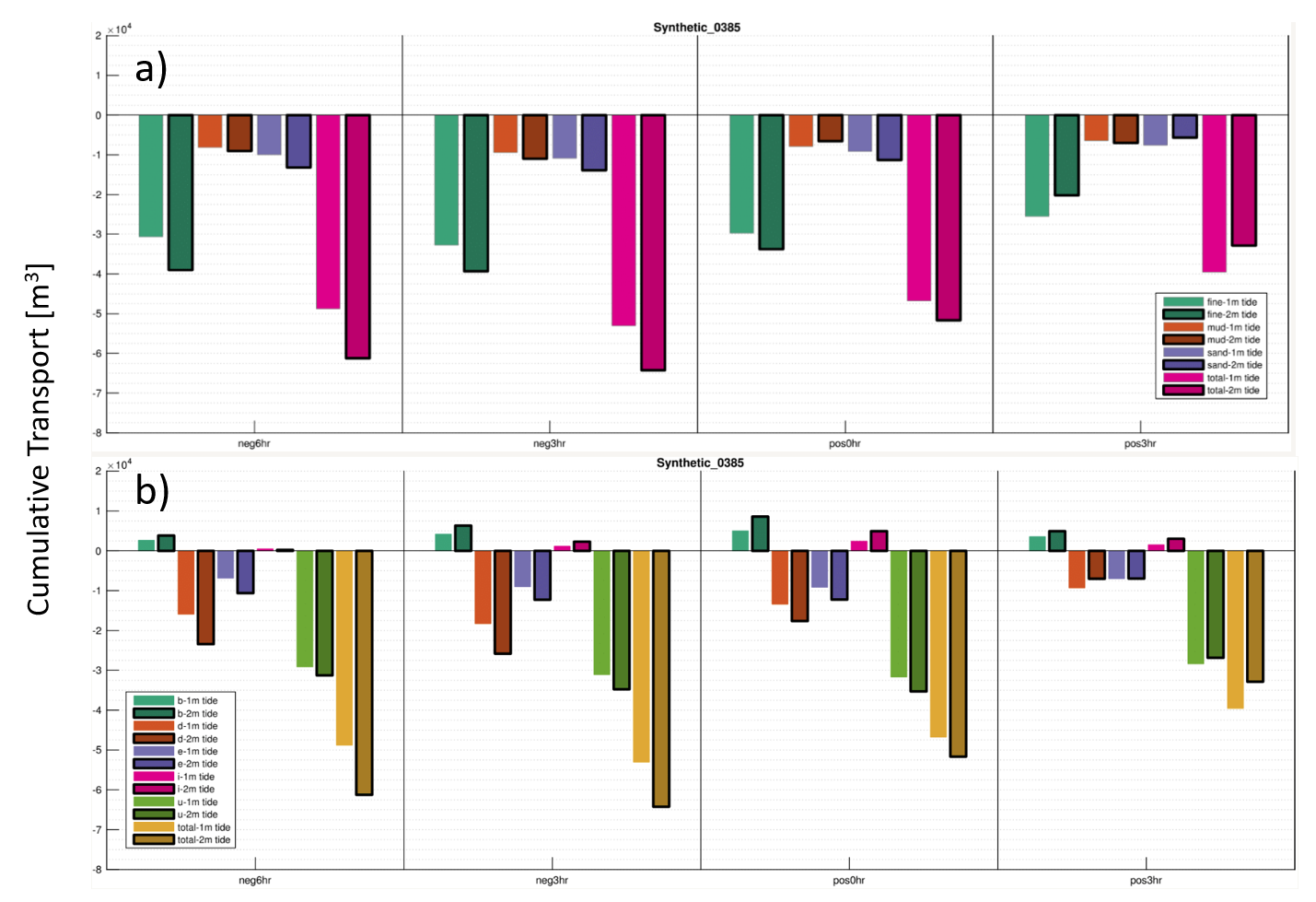


**Figure S18.** Cumulative sediment flux resulting from tidal range sensitivity analysis experiments assessing the influence of tidal amplitude on transport magnitude and direction at 1 m and and 2 m (all other storm scenarios used a tidal amplitude of 1.5 m). a) Transport for Storm 0385 shows that with tidal amplitude reduced to 1 m, the storm still produces sediment import but the import volumes are slightly lower compared to a tidal amplitude of 1.5 m. Likewise, when the tidal amplitude increased from 1.5 m to 2 m, sediment import volumes increase. For mud, import volumes vary only slightly across tidal amplitudes and tidal phasing and generally exhibit small (~1-3%) changes. Sand import volumes show slightly higher variability across tidal amplitude and tidal phasing experiment with up to 3-5% differences. Finally, fine sand exhibits the largest changes on order of 9-14%. The sensitivity analysis for tidal range shows no change in direction (all scenarios show net import) and little change in magnitude. Thus. we conclude storms import coarse sediment. The trends for tidal phasing are similar across all tidal ranges examined, with lower import volumes when the tidal peak precedes the surge peak by 3 hr (pos3hr) and higher import volumes when the tidal peak lags the surge peak by 6 hrs (neg6hr). b) Import volumes by sediment provenance for lower (1 m) and higher (2 m) tidal amplitude (see Figure 5 of main manuscript for zone delineation). Sediment import/export by provenance shows similar trends as the original experiments with tidal amplitude (1.5 m), with the import volumes modulated proportional to the tidal amplitude (i.e. volumes decrease slightly with lower tidal amplitude, and slightly increase with higher tidal amplitude).


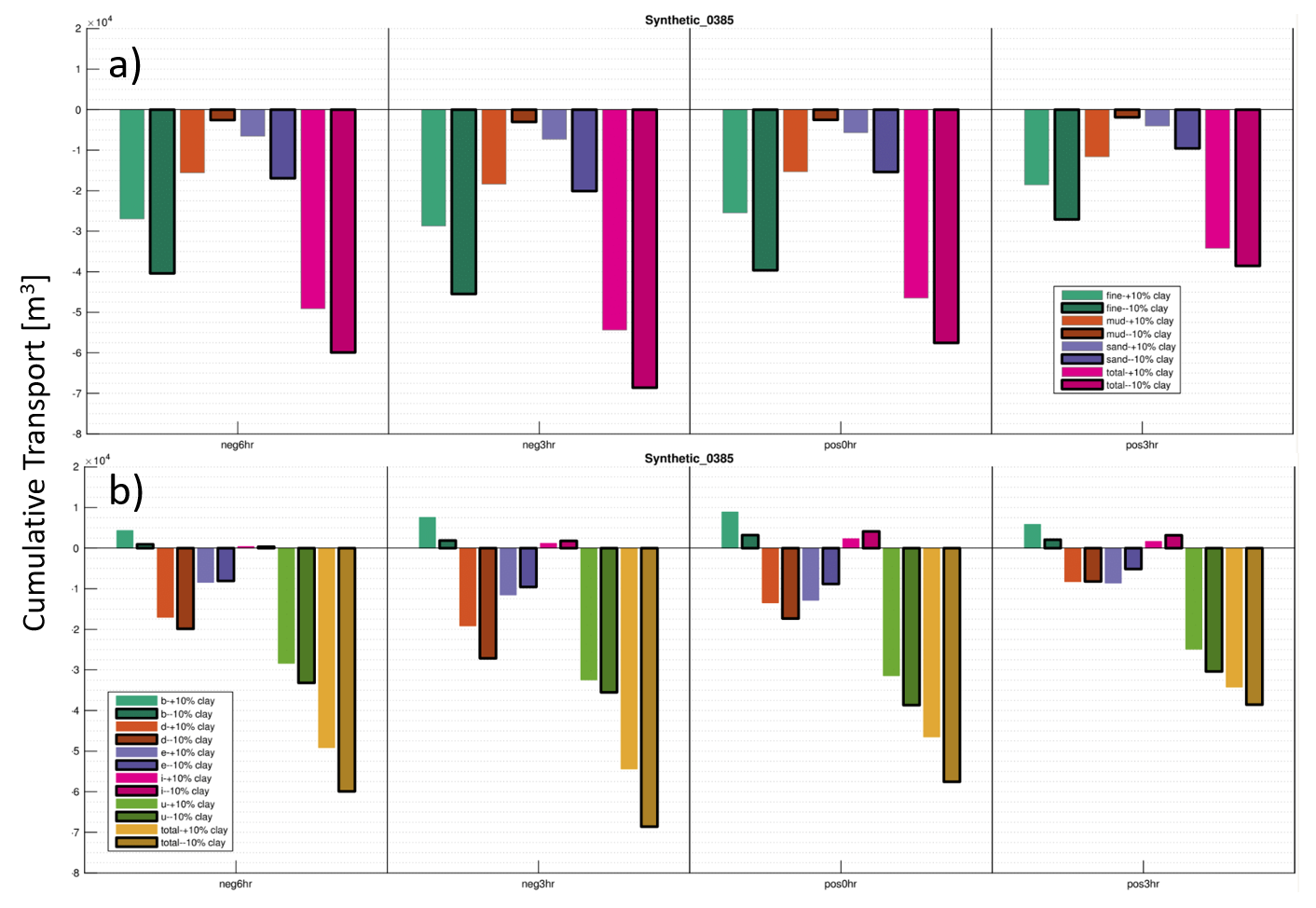


**Figure S19**. Cumulative sediment flux resulting from changes in clay content on import/export sediment volumes when reducing clay by 10% and when increasing by 10%. a) Transport for Storm 0385 shows that when the clay content is reduced from 14% to 4%, the storm still produces net import of sediment for all storms and all tidal phasing scenarios. However, the import volumes are slightly lower compared to the original clay content (14%), and when the clay content increased from 14% to 24%, net import volumes also increase. As expected, the analysis shows that mud transport is strongly influenced the most by adjusting clay content across tidal phasing scenarios. A 10% increase in clay content (from 14 to 24%) can increase import mud volumes by as much as 50%, whereas a decrease in clay content (from 14% to 4%) reduces mud import by 75%. Sand import volumes are inversely proportional to trends in mud and are reduced by 50% and increased by up to 60%, respectively. This is an expected outcome, since the adjustment in clay content, is at expense of sand. Fine sand import volumes varied less and exhibited similarly small (~10-25%) increase or decrease in import volumes. The trends for tidal phasing are similar across all clay content simulations with lower import volumes when the tidal peak precedes the surge peak by 3 hr (pos3hr) and higher import volumes when the tidal peak lags the surge peak by 6 hrs (neg6hr). Overall, the results are very similar and support the conclusion that storms import sediment. b) Import volumes by sediment provenance for lower (10% reduced) and higher (10% increased) clay content (see Figure 5 of main manuscript for zone delineation). Sediment import/export by provenance shows very similar trends as the original experiments with clay content of 14%, with the import volumes modulated proportional to the clay content and across tidal phasing scenarios.

**Table 1.** Mixed-Energy Barrier Island-Tidal Inlet Chains^9^

| Barrier System | Length of the Tidal Basin (Inlet to drainage divide, km) | Spring Tidal Range (m) |
| --- | --- | --- |
| Merrimack Embayment, MA | 8 – 12 | 3.3 |
| Western Long Island, NY | 7 – 17 | 2.0 |
| South New Jersey | 4 – 9 | 1.9 |
| Northern Virginia | 4 – 10 | 1.6 |
| Southwest North Carolina | 3 – 11 | 1.9 |
| Northern North Carolina | 3 – 7 | 2.1 |
| Southern South Carolina | 3 – 14 | 2.3 |
| Georgia | 7 – 15 | 2.5 |
| Northern Florida | 6 – 13 | 1.9 |
| West Frisian Islands | 10 – 20 | 2.4 |
| East Frisian Islands | 4 – 11 | 3.1 |
| Denmark | 11 – 22 | 2.3 |
| Algarve, Portugal | 4 – 5 | 3.1 |
| Copper River Delta, AK | 6 – 12 | 4.0 |

**SI References**

1. FitzGerald, D. M., Hughes, Z. J., Georgiou, I. Y., Black, S. & Novak, A. Enhanced, Climate-Driven Sedimentation on Salt Marshes. *Geophysical Research Letters* **47**, e2019GL086737 (2020).

2. Ioannis, Georgiou. Testing the runaway transgression hypothesis - Modeling Interactions between Backbarrier Marshes, Tidal Inlets, Ebb-deltas, and Adjacent Barriers Exposed to Rising Sea Levels. (2016).

3. Zhao, L. *et al.* Wetland-estuarine-shelf interactions in the Plum Island Sound and Merrimack River in the Massachusetts coast. *Journal of Geophysical Research: Oceans* **115**, (2010).

4. Wilson, C. A. *et al.* Saltmarsh pool and tidal creek morphodynamics: Dynamic equilibrium of northern latitude saltmarshes? *Geomorphology* 17 (2014) doi:10.1016/j.geomorph.2014.01.002.

5. Zoe, Hughes, Duncan, M, FitzGerald & Ioannis, Y. Georgiou. Assessing the extent and Importance of sedimentation from a 100-year winter storm on salt marsh resilience. (2018).

6. Hopkinson, C. S., Morris, J. T., Fagherazzi, S., Wollheim, W. M. & Raymond, P. A. Lateral marsh edge erosion as a source of sediments for vertical marsh accretion. *Journal of Geophysical Research: Biogeosciences* **123**, 2444–2465 (2018).

7. Zhang, X., Leonardi, N., Donatelli, C. & Fagherazzi, S. Fate of cohesive sediments in a marsh-dominated estuary. *Advances in Water Resources* **125**, 32–40 (2019).

8. Mukai, A. Y., Westerink, J. J., Luettich, R. A. (Richard A. & Mark, D. J. *Eastcoast 2001, a tidal constituent database for the Western North Atlantic, Gulf of Mexico, and Caribbean Sea*. *This Digital Resource was created from scans of the Print Resource.* https://erdc-library.erdc.dren.mil/jspui/handle/11681/7521 (2002).

9. Hanegan, K. C., FitzGerald, D. M., Georgiou, I. Y. & Hughes, Z. J. Long-term sea level rise modeling of a basin-tidal inlet system reveals sediment sinks. *Nat Commun* **14**, 7117 (2023).
